# Detection of the Carcinogen Benzo[*a*]pyrene through Photochemically Induced Dynamic Nuclear Polarization: Linking Liquid-State ^1^H NMR with Spatially Resolved Imaging

**DOI:** 10.1021/acs.analchem.6c00224

**Published:** 2026-04-29

**Authors:** Mohd Humair Ali, Guzel Musabirova, Luca Gerhards, Ilia A. Solov’yov, John P. Berry, Jörg Matysik, A. Alia

**Affiliations:** † Institut für Analytische Chemie, 9180 Universität Leipzig, Linnéstr. 3, Leipzig D-04103, Germany; ‡ Institut für Medizinische Physik und Biophysik, Universität Leipzig, Härtelstr.16-18, Leipzig D-04107, Germany; § Institute of Physics, 11233 Carl von Ossietzky Universität, Carl von Ossietzky Str. 9-11, Oldenburg D-26129, Germany; ∥ Center for Nanoscale Dynamics (CENAD), Carl von Ossietzky Universität, Ammerländer Heerstr. 114-118, Oldenburg D-26129, Germany; ⊥ Research Centre for Neurosensory Science, Carl von Ossietzky Universität, Carl-von-Ossietzky-Str. 9-11, Oldenburg D-26129, Germany; # Department of Chemistry and Biochemistry, Florida International University, 3000 NE 151st Street, North Miami, Florida 33199, United States; ∇ Leiden Institute of Chemistry, Leiden University, Einsteinweg 55, Leiden 2301 RA, The Netherlands

## Abstract

Benzo­[*a*]­pyrene (BaP), a polycyclic aromatic hydrocarbon (PAH) with significant environmental prevalence and toxicological implications, is classified as a group 1 carcinogen. Nonetheless, the noninvasive *in vivo* traceability of BaP in biological systems faces a major challenge. In this study, we introduce a novel noninvasive NMR and MRI detection method for BaP utilizing light-induced signal enhancement through the photochemically induced dynamic nuclear polarization (photo-CIDNP) effect to achieve new limits in sensitivity, contrast, and spatial resolution. Liquid-state ^1^H NMR revealed a substantial photo-CIDNP signal enhancement of BaP in the presence of riboflavin, as a photosensitizer, under illumination, corroborating the hyperpolarization phenomenon. Furthermore, BaP signal enhancement in dodecylphosphocholine (DPC) micelles, a biological membrane-simulating environment, demonstrated the viability of employing photo-CIDNP in intricate, heterogeneous biological systems. The approach was subsequently expanded to MRI-based chemical shift imaging (CSI) to spatially map the radical-polarized signals from encoded voxels. The mapping of CSI images demonstrated that light-induced signals were confined to regions where the light was projected, hence affirming the spatial correlation between light illumination and increased signal intensity, laying a foundation for its potential as a diagnostic tool in ^1^H MRI. This study provides a viable application of ^1^H photo-CIDNP MRI as a sensitive approach that can be applied for monitoring environmental toxins noninvasively and establishes a robust link between photochemistry and *in vivo* molecular-level MRI visualization.

## Graphical abstract

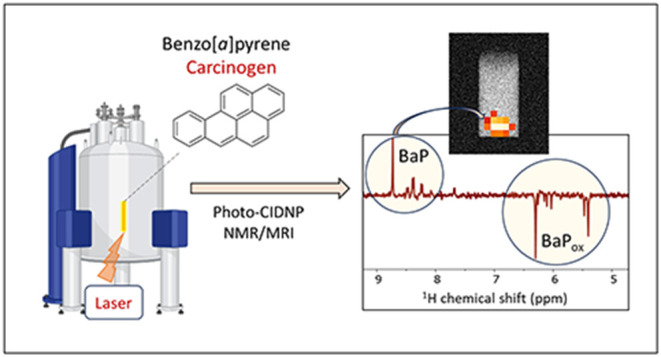

## Introduction

Benzo­[*a*]­pyrene (BaP) is a polycyclic aromatic hydrocarbon (PAH) classified as a Group 1 human carcinogen by the International Agency for Research on Cancer (IARC), owing to its well-established carcinogenicity and widespread environmental presence.^1^ It is primarily introduced into the environment via incomplete combustion processes, including industrial emissions, vehicular exhaust, and biomass burning.^2^ Human exposure occurs through inhalation of contaminated air, dermal absorption from contaminated surfaces,^3^ or ingestion of polluted food and water.^4^ A major contributor to BaP exposure is tobacco smoke, wherein sidestream concentrations range from 52 to 95 ng per cigarette over 3-fold higher than those detected in mainstream smoke. Following systemic absorption, BaP undergoes metabolic activation via the cytochrome P450 enzyme, leading to the formation of 7β, 8α-dihydroxy-9α, 10α-epoxy-7, 8, 9, 10-tetrahydrobenzo­[*a*]­pyrene (BPDE), which forms covalent adducts with DNA, induces mutagenesis, and initiates oncogenic transformation.^5^ Furthermore, several oxidized BaP species including epoxides, dihydrodiols, phenols, and quinones can be formed enzymatically *in vivo*, leading to carcinogenic aberrations.^6^

Beyond its well-characterized carcinogenicity, BaP and its oxidative derivatives cause epigenotoxic, neurotoxic, and teratogenic effects, while also contributing to reproductive toxicity and oxidative stress.^7^ The toxic level of BaP in the serum and in other biological fluids has been found to be only in the nanogram range (0.4–2 ng/mL).^8,9^ The level of its bioactive intermediate, BPDE, was detected as a protein adduct in the range of 8.87–33.55 ng/mL in smoker, indicating an increased burden of BaP-derived genotoxic intermediates.^10^ It is likely that BaP and its bioactive metabolites accumulate over time in various tissue and cell compartments. However, noninvasive *in vivo* detection of BaP in various tissues and organs is not yet possible.

While magnetic resonance methods such as nuclear magnetic resonance (NMR) offer exclusive chemical specificity and magnetic resonance imaging (MRI) can allow noninvasive detection of the metabolites in localized regions *in vivo*, the physiologically relevant concentrations of BaP are orders of magnitude below the detection limits of conventional NMR and MRI, owing to its inherently low sensitivity, limited by the Boltzmann distribution.^11^ However, these limitations can be mitigated by hyperpolarization techniques that selectively enhance specific peaks in the NMR spectrum and enable the detection of low-abundance biologically relevant molecules.

Photo-Chemically Induced Dynamic Nuclear Polarization (photo-CIDNP) is an advanced hyperpolarization method in NMR in which spin-correlated radical pairs (SCRPs) are created upon illumination with visible light. In liquid-state NMR, the enhancement is often due to the classical radical pair mechanism (RPM), relying on spin-sorting and requiring free diffusion.^12^ Photo-CIDNP NMR has been successfully applied to various amino acids (e.g., tryptophan, tyrosine, histidine, methylated lysines, and methionine)^13^ and protein systems (e.g., flavoproteins, bovine alpha-lactalbumin, trypsin inhibitors, phosphoglycerate kinase, and calf-gamma II Crystallin).^14,15^ The use of photo-CIDNP NMR in detecting toxins, such as BaP and its metabolic intermediates, has not been demonstrated so far.

Despite their potential, photo-CIDNP techniques remain largely unexplored in the context of MRI. MRI is a versatile and noninvasive imaging technique widely used in both clinical and research settings due to its excellent spatial resolution and soft tissue contrast. However, its primary limitation is low sensitivity, stemming from the weak magnetic signals generated by nuclei under thermal polarization, which restricts its ability to detect low-concentration biomarkers or subtle molecular changes. The production of spin-hyperpolarization, providing non-Boltzmann spin populations, can overcome this problem. Several hyperpolarization methods, such as dynamic nuclear polarization (DNP),^16–25^ spin-exchange optical pumping (SEOP),^26–33^ and para-hydrogen-induced nuclear polarization (PHIP),^34–37^ have been applied to MRI. The photo-CIDNP hyperpolarization method may offer a promising approach to enhancing nuclear spin polarization using light-driven processes and can provide significant signal enhancements in MRI. They operate under mild conditions without the need for cryogenics or radical introduction and allow for rapid, repeatable polarization cycles, making them attractive candidates for future adaptation to MRI. Photo-CIDNP-enhanced MRI has previously been demonstrated in systems involving ^19^F nuclei, using 3-fluoro-dl-tyrosine,^38,39^ and the fluorinated drug favipiravir.^40^ However, to date, ^1^H photo-CIDNP MRI has been reported in only a single study, using a standard solution of 2,2-dipyridyl and *N*-acetyl tryptophan system.^41^

As a polycyclic aromatic hydrocarbon, BaP may exhibit a photo-CIDNP effect via a radical pair mechanism, acting as an electron donor in the process. The aim of this study was to investigate whether BaP, in combination with the photosensitizer riboflavin (RF), can undergo photo-CIDNP and yield enhanced signal intensities in liquid-state ^1^H NMR experiments, which would establish the feasibility of detecting BaP via light-induced nuclear spin hyperpolarization. We first analyzed the effect of the concentration of BaP and the magnetic field strengths, which could offer optimum photo-CIDNP conditions for the BaP and RF system. Subsequently, the BaP and RF system was incorporated into DPC micelles, mimicking a biological membrane-like environment, to study whether signal enhancement of BaP under photo-CIDNP can occur in a setting that more closely reflects physiological conditions. In addition, the photo-CIDNP MRI method was employed, for the first time, to spatially map the radical-polarized signal from BaP using chemical shift imaging. This novel approach represents a pioneering attempt to bridge molecular spin-dynamics with imaging and could lay the groundwork for photoresponsive MRI contrast methods in biologically relevant systems.

## Experimental Methods

### Chemicals

BaP was purchased from Supelco, United States. All other chemicals were purchased from Sigma-Aldrich (St. Louis, MO, USA) unless otherwise stated.

### Sample Preparation

For liquid-state ^1^H photo-CIDNP NMR experiments, a 10 mM stock solution of BaP was prepared in dimethyl sulfoxide-*d*_6_ (DMSO-*d*_6_, 99.9%). Fresh 2 mM RF solutions in DMSO-*d*_6_ were prepared prior to each ^1^H NMR and ^1^H-MRI experiment. For ^1^H photo-CIDNP NMR measurements, samples were prepared by mixing BaP and RF in DMSO-*d*_6_ such that the RF concentration was kept constant at 0.2 mM, while BaP concentrations were adjusted to achieve BaP:RF in ratios of 1:1, 2:1, 3:1, 5:1, 7:1, and 10:1. The 5:1 ratio, selected based on the photo-CIDNP efficiency of the system, was further used for ^1^H photo-CIDNP NMR of the DPC-based micellar system and for MRI experiments.

For mimicking the biological membrane environment, BaP (1 mM), DPC (5 mM), and DPC-*d*_38_ (10 mM) were dissolved in ∼10 mL of chloroform. A homogeneous thin film of BaP-incorporated DPC micelles was obtained upon evaporation of chloroform using a rotary evaporator at 313.15 K, 130 mbar, and 120 rpm. A mixture of protonated and deuterated DPC was used to exceed the critical micelle concentration while minimizing the background proton signals in the ^1^H NMR spectra. The final mixture of BaP, RF, DPC, and DPC-*d*_38_ was prepared by adding an aqueous solution of RF (0.2 mM) in D_2_O to the dry film of micelles. The mixture was gently vortexed and equilibrated for 15 min at room temperature.

### Illumination Setup

For liquid-state ^1^H photo-CIDNP NMR, a 445 nm diode laser (MDL-III-445-1000 mW, Ultralasers Inc., Canada; output power: 900 mW, multimode, TTL-modulated) was used for sample illumination. The laser beam was guided by a light fiber (1000 μm, FP1000URT-0.5 NA) from Thorlabs (Bergkirchen, Germany) into the solution via a glass insert (5-BL, Wilmad, Vineland, NJ) that could be lowered in the NMR tube (Figure 1A). The laser was operated in modulation mode and triggered directly by the NMR console. The TTL signal from the console was routed through an inverter, enabling the precise synchronization of light irradiation with the pulse sequence (Figure 1B). For ^1^H photo-CIDNP MRI, a 445 nm, 600 mW laser beam was delivered through an optical fiber inserted at the bottom of the 10 mm NMR tube to ensure localized illumination of the sample. The laser was used in continuous wave mode. The setup enabled uninterrupted irradiation throughout the CSI acquisition, allowing for consistent light-induced polarization effects across the spatially resolved voxels.

**Figure 1:**
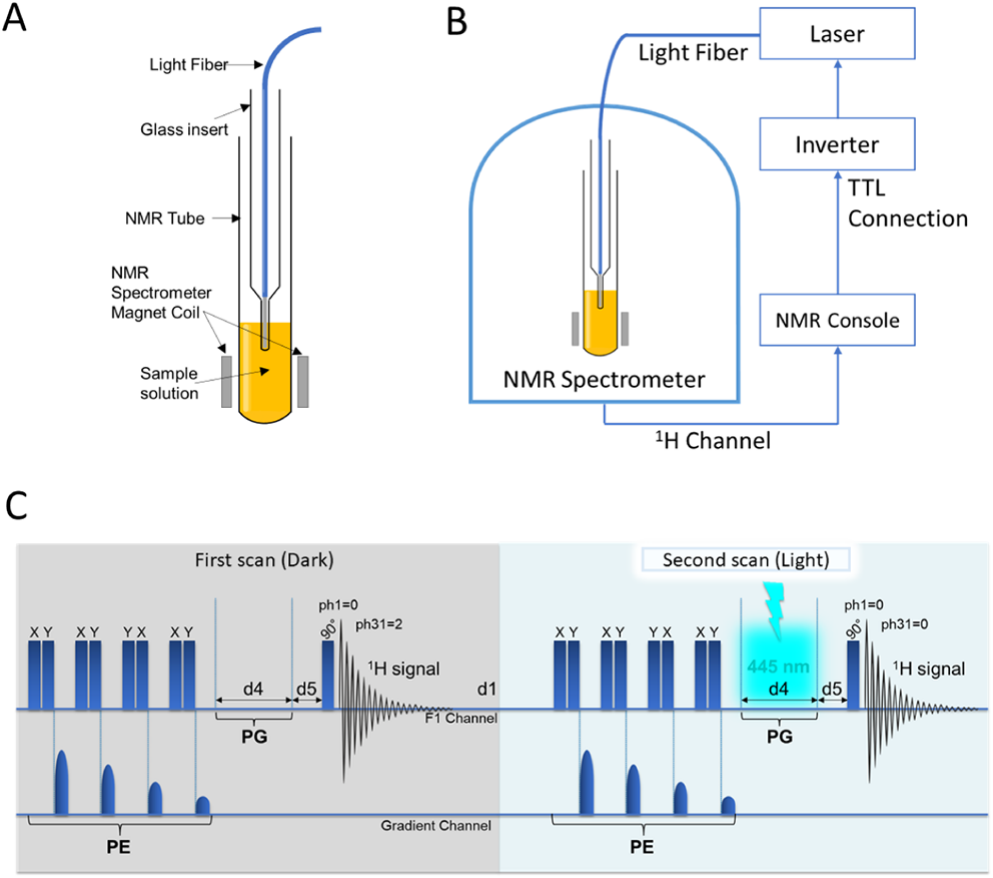
Illumination setup and pulse sequence used for the photo-CIDNP NMR studies. (A) NMR tube with a glass insert submerged in the solution; (B) Schematic representation of the photo-CIDNP setup. A fiber was inserted into the NMR tube containing the liquid sample, enabling the delivery of light directly into the NMR spectrometer. The laser was triggered by the NMR console via an inverter, ensuring synchronization between the light flash and the NMR pulse sequence; (C) Pulse sequence used for ^1^H photo-CIDNP NMR containing a presaturation pulse train (polarization extinction (PE) block) designed for background suppression prior to optical excitation, as described previously.^42^ A destructive phase cycle with irradiation in every second scan was used to acquire the ^1^H photo-CIDNP NMR spectra.

### Liquid-State ^1^H Photo-CIDNP NMR

^1^H photo-CIDNP NMR measurements of BaP and RF in DMSO-*d*_6_ were performed at 400 and 600 MHz (Bruker Biospin GmbH, Germany) equipped with 5 mm Broadband Inverse (BBI) and Triple resonance Broadband Inverse (TBI) probes, respectively. ^1^H photo-CIDNP NMR measurements of BaP and RF in a DPC-based micellar system were performed on a 400 MHz spectrometer. Manual probe tuning and matching (wobbling) were performed before each experiment to ensure optimal radiofrequency (rf) performance. All measurements were conducted at 293 K using a Bruker BVT3000 control unit.

The pulse sequence used for ^1^H photo-CIDNP NMR is shown in Figure 1C. The sequence comprises a polarization extinction block that includes a presaturation pulse train followed by a z-gradient to suppress the formation of spin echoes.^42^ Presaturation was used to eliminate all of the initial Boltzmann polarization in the sample prior to optical excitation. To avoid errors associated with subtracting light and dark spectra acquired from separate experiments, photo-CIDNP difference spectra were recorded directly by using a destructive phase cycle, in which laser irradiation was applied in every second scan to directly generate dark-minus-light ^1^H photo-CIDNP difference spectra.^42^

Each ^1^H photo-CIDNP NMR spectrum was acquired with 32 scans, alternating between 16 scans with illumination and 16 scans without illumination, with a laser exposure duration of 0.5 s per illuminated scan. A multiscan acquisition, 10 repeats, was conducted, and the final ^1^H photo-CIDNP NMR spectrum was obtained by summing up all the 10 experiments.

All spectra were acquired using a spectral width of 12000 and 8000 Hz at 600 and 400 MHz, respectively, a receiver gain of 101, an artificial line broadening of 2 Hz, a recycle delay of 4 s, and a delay after illumination of 0.5 s.

### DFT Calculations

All quantum-chemical calculations were performed with the ORCA program package (version 6.1).^43^ Geometry optimizations were carried out using the UKS B3LYP-D4 method^44–46^ with the RIJCOSX approximation^47^ and the pcJ-3 basis set.^48^ Solvent effects of DMSO were included employing the CPCM solvation model.^49^ Tight convergence criteria were applied for both the SCF procedure (TightSCF) and geometry optimization (TightOpt). The SOMF­(1X) approximation was used for spin–orbit mean-field effects. Hyperfine coupling constants and g-tensors were obtained from single-point calculations at the optimized geometries using the EPRNMR module of ORCA. For the hyperfine couplings, the set of all hydrogen nuclei was explicitly included (Nuclei = ALL H {AISO, ADIP, Rho, AOrb}), ensuring the calculation of isotropic Fermi-contact terms, dipolar contributions, spin density at the nucleus, and orbital contributions. g-tensor calculations were performed with the GTensor True keyword.

### Photo-CIDNP MRI

^1^H photo-CIDNP MRI experiments of BaP and RF in DMSO-*d*_6_ were performed using a Bruker 300 MHz vertical bore NMR system (Bruker Biospin GmbH, Germany). A birdcage transmit/receive rf coil with an internal diameter of 10 mm was used. The workstation was linked to a Linux operating system running ParaVision 5.1 imaging software (Bruker Biospin GmbH, Germany). Prior to data acquisition, magnetic field homogeneity was optimized by shimming, followed by resonance frequency calibration and adjustment of the transmitter and receiver gains. All measurements were performed at 293 K. For MRI, a solution of BaP and RF at a ratio of 5:1 was used.

Each measurement began with a multislice orthogonal gradient-echo sequence to ascertain the position of the sample and select the desired region. A rapid acquisition with relaxation enhancement (RARE) sequence^50^ was used to acquire a reference image. RARE generates multiple rf spin echoes, which allows faster image acquisition by acquiring more than one k-space line per repetition.^50^ The measurement parameters used for the RARE sequence were as follows: Echo time (TE) = 8.5 ms with an effective echo time of 18.06 ms; Repetition time (TR) = 1000 ms; Number of scans (ns) = 1; Total scan time = 128 s; RARE factor = 4. The field of view (FOV) was 4 cm with a matrix size of 256 × 256. The slice thickness was 4 mm.

Multivoxel MR spectroscopic information was acquired using CSI in the spin-echo slab-selective mode. CSI employs two orthogonal phase-encoding steps with pulsed gradients, capturing a pure spectroscopic echo during acquisition, distinct from the conventional readout gradient used in imaging.^51^ Spatially resolved multivoxel spectra were obtained through the simultaneous acquisition of spatial and spectral information, resulting in spatial distribution maps. The spin-echo sequence with 90° and 180° pulses and the stepped phase-encoding gradient pulses in the *x* and *y* directions identified the pixel location. The bandwidth of water suppression rf pulses was narrow and centered on the resonance frequency of the water peak to saturate the water signal. The measurement parameters were as follows: TE = 15 ms; TR = 1000 ms; Matrix = 32 × 32; FOV = 40 × 40 mm^2^; Slice thickness = 4 mm; ns = 256. Data were reconstructed into a 32 × 32 matrix with linear smoothing for display purposes. For excitation and refocusing, Sinc3 pulses with a bandwidth of 5645.5 Hz were used. A spectral width of 8 kHz and a spectral resolution of 1.96 Hz per point were obtained from the acquisition of echoes in 2048 points over 255.59 ms. Water resonance shimming was used to achieve the optimum magnetic field homogeneity in the selected volume. A Variable Power RF pulses with Optimized Relaxation delays (VAPOR) suppression scheme of 625 ms duration was applied to effectively saturate the water signal. Seven Hermite-shaped CSI modules with interpulse rf delays of 150, 80, 160, 80, 100, 37.2, and 15 ms were used. The excitation offset was −75 Hz (−0.1 ppm), and the rf bandwidth was 900 Hz. The CSI spectra at each voxel and mapping of light-induced signals were performed using the CSI visualization tool of the ParaVision 5.1 imaging software (Bruker Biospin GmbH, Germany).

## Results and Discussion

### ^1^H Photo-CIDNP NMR of BaP

The first objective was to determine whether BaP exhibits photo-CIDNP effects upon optical excitation in the presence of an RF photosensitizer. The ^1^H photo-CIDNP NMR spectra of BaP and RF under 445 nm laser illumination, measured using a phase-destructive pulse sequence, are shown in Figure 2A. The figure illustrates that the photo-CIDNP spectrum of BaP exhibits absorptive polarization for nearly all protons. The signal-to-noise ratios under illumination were approximately 49 (H-6), 15 (H-1 and H-7), 8 (H-12), and 11 (H-3). The ^1^H NMR signal assignments for BaP were confirmed by 2D COSY and ^1^H–^13^C heteronuclear single quantum coherence (HSQC) experiments (see Supplementary Figure S1). The photo-CIDNP pattern of the RF proton is consistent with previously reported data for isoalloxazine systems.^42,52–54^

**Figure 2:**
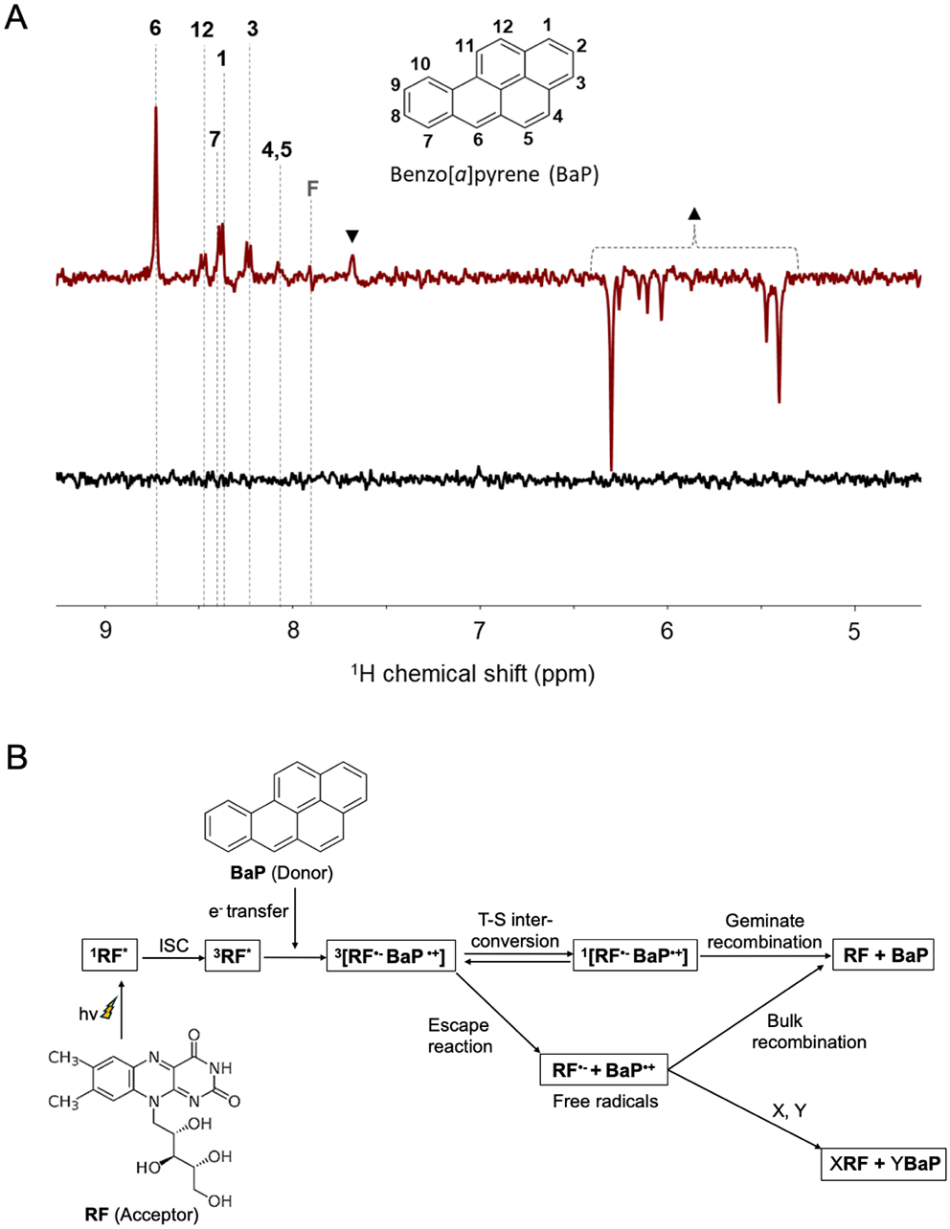
(A) ^1^H photo-CIDNP NMR spectra of BaP (1 mM) and RF (200 μM) in DMSO-*d*_6_, acquired at 400 MHz and 293 K, were recorded with polarization extinction under 445 nm (900 mW) laser illumination (spectrum red) and in the dark (spectrum black). Assignments of specific proton resonances of BaP are indicated, highlighting the hyperpolarization effect in the photo-CIDNP spectrum. A signal attributed to the photodegradation product of RF appears at 7.7 ppm and is marked with down-triangle (▼). Signals ranging from 5.4 to 6.3 ppm, observed exclusively under illumination from the oxidative product of BaP, not detectable in the dark, are marked with up-triangle (▲). (B) Possible reaction pathways involving the radical pair mechanism in the photo-CIDNP of BaP and the RF system.

The origin of the observed liquid-state photo-CIDNP might be explained by the classical radical-pair mechanism (RPM),^39,55^ occurring upon radical-pair formation with BaP as an electron donor and RF as an electron acceptor (Figure 2B). Upon illumination, RF is first transferred to its excited singlet state and then undergoes intersystem crossing to a triplet state, where it subsequently engages light-induced electron transfer from BaP, resulting in the generation of a triplet-born radical pair. In an aerated environment, the RF triplet can also transfer energy to ^3^O_2_ to produce singlet oxygen (^1^O_2_, type-II pathway), which competes with electron transfer and can diminish the radical-pair yield.

In accordance with Kaptein’s sign rule (Γ = μ·ε·Δ*g*·a),^56^ the observed photo-CIDNP effect from BaP implies that the radical pair is formed from a triplet precursor (μ > 0) with geminal recombination dominating over escape products (ε > 0). The density functional theory (DFT) calculations (see Supplementary Table S1) yielded negative hyperfine coupling constants (HFCCs) (*a* < 0) almost for all BaP protons and a Landé *g*-factor value of 2.0026, smaller than the RF *g*-factor (Δ*g* < 0). These parameters predict absorptive polarization for protons H-1, H-3, H-4, H-5, H-6, H-7, H-9, H-10, and H-12, and emissive polarization for protons H-2, H-8, and H-11 of BaP.

The magnitude of the observed photo-CIDNP enhancement in BaP generally follows the trend of the HFCCs. DFT calculations were performed to determine the HFCCs, and the results indicate that protons H-6, H-12, H-1, and H-3 show strong positive signals due to relatively large HFCCs, while H-2, H-5, and H-4 exhibit weaker effects, consistent with smaller couplings. Protons H-10 and H-11 were not polarized, in line with their near-zero HFCCs (−0.68 and 0.12 MHz, respectively). The expected positive polarization of H-9 is not observed because its resonance overlaps with those of H-8 and a flavin proton, both showing emissive polarization, resulting in apparent signal cancellation in the CIDNP spectrum (Figure 2A).

Interestingly, in the photo-CIDNP NMR spectra, we observe additional intense emissive signals within the δ_H_ ≈ 5.4–6.3 ppm that cannot be assigned to either BaP or RF (Figure 2A). The signal-to-noise ratios of the emissive signals under illumination were approximately 60 at 6.3 ppm and 36 at 5.35 ppm. To rationalize these signals, it is important to consider the oxidative behavior of BaP. BaP is known to undergo oxidation to yield various oxygenated derivatives, including epoxides, dihydrodiols, phenols, and quinones. According to the study by Miller and Ramos,^6^ several oxidized BaP species can be formed enzymatically *in vivo*. In our system, however, illumination of RF might facilitate the formation of singlet oxygen (^1^O_2_) through energy transfer from the excited triplet state of flavin to triplet molecular oxygen. It has been demonstrated that singlet oxygen can oxidize BaP to its derivatives in the absence of enzymes, leading to the formation of mutagenic products.^57^ Previously, Lee-Ruff et al.^58^ showed that quinone derivatives can be produced from BaP through both photo-oxidation and singlet-oxygen-mediated reactions. Thus, it is likely that the unassigned emissive photo-CIDNP signals observed in Figure 2A originate from one or more photo-oxidation products of BaP. The emissive peaks appear only in the photo-CIDNP spectra and increase in intensity with extended irradiation time, as shown in Supplementary Figure S3. This temporal growth indicates progressive formation of the photogenerated oxidation products within the sample during the measurement, consistent with their accumulation under continued light exposure. These emissive photo-CIDNP signals were not observed when the BaP-RF solution was degassed by nitrogen bubbling prior to illumination (Supplementary Figure S4), consistent with an oxygen-dependent oxidative process.

To identify the origin of these additional emissive signals, we examined a range of possible BaP oxidation products (see Supplementary Figure S5) and selected several candidates that might cause proton resonances within the δ_H_ 5.4–6.3 ppm region. For these species, HFCCs were calculated using DFT (Supplementary Table S1 and Figure S2). According to Kaptein’s sign rules, emissive photo-CIDNP effects are expected for protons with positive HFCCs, and the signal intensity should scale with the magnitude of the coupling constant.^56^ Therefore, particularly large HFCC values are responsible for the observed signals of different species. Among the various diol and hydroquinone derivatives examined, benzo­[*a*]­pyrene-1,6-hydroquinone (H-1: 104.08 MHz and H-6: 63.67 MHz), benzo­[*a*]­pyrene-6,12-hydroquinone (H-6: 19.75 MHz and H-12: 128.25 MHz), and benzo­[*a*]­pyrene-3,6-hydroquinone (H-3: 76.72 MHz and H-6: 48.18 MHz) exhibited the strongest agreement with the observed emissive photo-CIDNP signals (see Supplementary Figure S5). The proposed photochemical pathway that rationalizes these observations is summarized by reactions (eqs 1–5), linking singlet-oxygen formation, BaP oxidation, radical-pair generation, and spin-selective back electron transfer. This sequence can be represented schematically as follows (Abbreviation: BaP_hq_= hydroquinone derivatives of BaP; BaP_ox_ = other possible oxidative products of BaP; ^1^O_2_/^3^O_2_ = singlet/triplet oxygen):

1 RF3+O23→RF+O21

2 O21+BaP→BaPhq+BaPox

3 RF3+BaPhq→[RF3•−⋯BaPhq•+]

4 [RF3•−⋯BaPhq•+]↔[RF1•−⋯BaPhq•+]

5 [RF1•−⋯BaPhq•+]→RF1+BaPhq

To check whether oxidation products were already present in small amount as contaminants or they are formed during illumination, we compared the thermally polarized ^1^H NMR spectra of BaP and RF, in the dark, before and after illumination, using a conventional single pulse (zg) sequence. No signals corresponding to hydroquinone derivatives of BaP were detected in the spectrum recorded with 32 scans (Supplementary Figure S6A) in the dark before and after illumination. However, when the number of scans was increased substantially (ns = 4096), signals from hydroquinone derivatives of BaP became detectable in the dark after illumination (Supplementary Figure S6B). Our results suggest that these oxidized derivatives are formed during illumination and are present in a very low amount. Signal-to-noise ratio and integration analyses indicated that the concentration of the photogenerated oxidation products was approximately 4 μM (1.1 μg/mL). Despite this low amount, photo-CIDNP spectra obtained with only 32 laser-modulated scans displayed pronounced emissive signal enhancements from these photogenerated oxidation products (Figure 2A). This dramatic optical amplification is fully consistent with the unusually large HFCCs predicted by DFT calculations for hydroquinone-type BaP derivatives bearing hydroxyl groups at nonadjacent positions (1,6-, 3,6-, and 6,12-substituted species), yielding couplings in the range of approximately 50–130 MHz. Strongly coupled protons are predicted to resonate within the δ_H_ 5.4–6.3 ppm region, in excellent agreement with the experimental photo-CIDNP observations (Supplementary Figure S5).

Since hydroquinone-type BaP metabolites are key oxidative intermediates in BaP metabolism,^58^ their detection via photo-CIDNP is biologically relevant, as these metabolites are known to take part in redox cycling and produce electrophilic species that can adduct DNA or generate reactive oxygen species, processes associated with BaP-induced mutagenesis and carcinogenesis.^59,60^ Thus, our work opens new opportunities to detect not only BaP but also its metabolites in low concentrations with photo-CIDNP signal enhancement in biologically relevant samples.

### Concentration Dependence of BaP in ^1^H Photo-CIDNP NMR

To analyze the efficiency of photo-CIDNP as a function of BaP concentration, ^1^H photo-CIDNP NMR spectra were acquired under illumination for BaP and RF at molar ratios of 1:1, 2:1, 3:1, 5:1, 7:1, and 10:1, while keeping the RF concentration constant (200 μM), in DMSO-*d*_*6*_ at 400 MHz (9.4 T), as shown in Figure 3. In the concentration range of BaP from 200 to 1400 μM, emissive signals were observed for RF protons at 7.9 ppm. However, upon an increase in the BaP concentration to 2000 μM, these RF signals gradually disappear. The loss of RF emissive signals at higher BaP concentrations likely results from overlap with oppositely phased BaP polarization.

**Figure 3:**
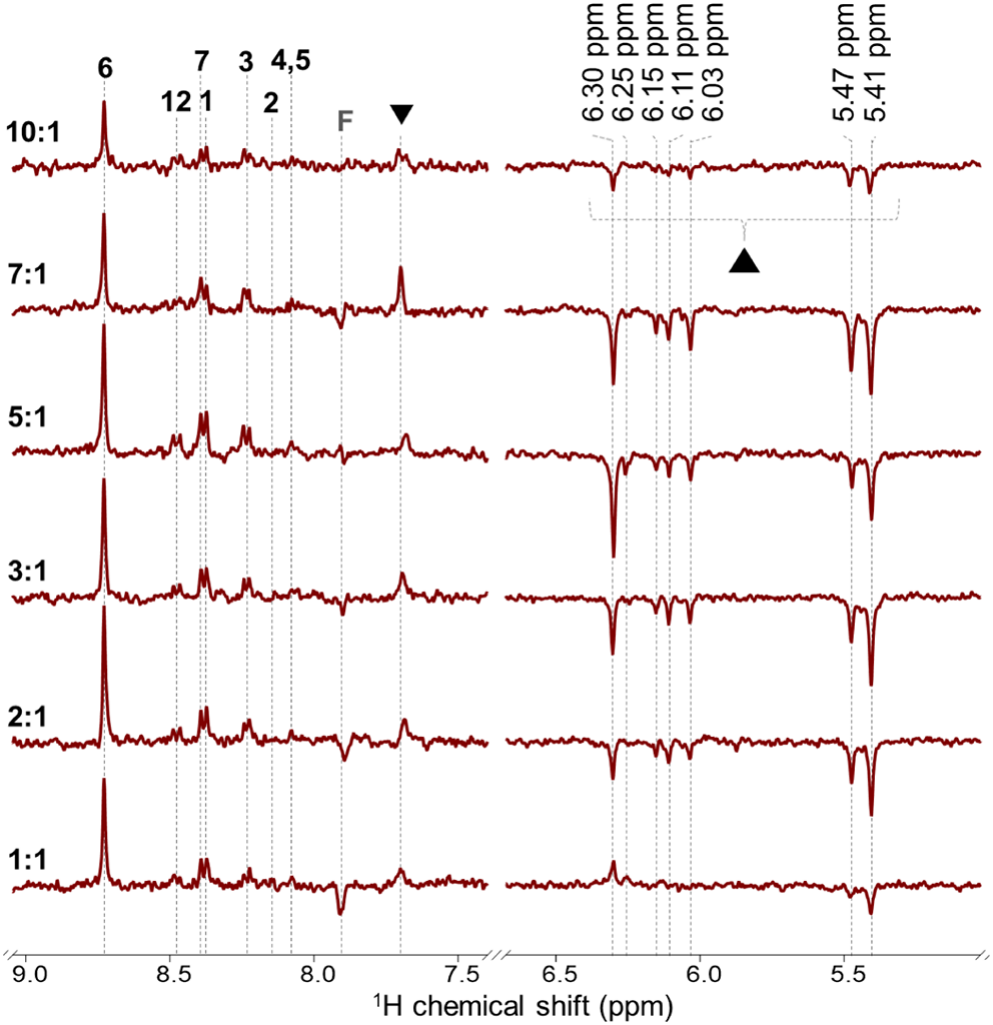
^1^H photo-CIDNP NMR spectra of BaP and RF (200 μM) in DMSO-*d_6_*, recorded at 400 MHz and 293 K under 445 nm laser illumination (0.9 W; 0.5 s per scan). Spectra are arranged bottom to top for BaP:RF molar ratios of 1:1, 2:1, 3:1, 5:1, 7:1, and 10:1. Signal assignments correspond to the proton numbering in the molecular structure of BaP (see Figure 1). A signal attributed to the photodegradation product of RF appears at 7.7 ppm and is marked with down-triangle (▼). Signals at 5.4–6.3 ppm, observed exclusively under illumination from the oxidative product of BaP, are marked with up-triangle (▲).

It is intriguing that overall photo-CIDNP signal intensities of BaP do not continue to increase as anticipated at a 10:1 ratio (Figure 3 and Supplementary Figure S7). On the contrary, a slight reduction in the signal intensities is observed when compared with intermediate ratios (2:1, 3:1, 5:1, and 7:1). Furthermore, the emissive photo-CIDNP signals at 5.4–6.3 ppm, hypothetically assigned to BaP_hq_, show a similar photo-CIDNP intensity trend as BaP. For all species, the polarization of BaP first grows with increasing BaP concentration due to the increase in the quantum yield of radical pair formation, which competes more effectively with other ^3^RF decay channels. Further increase in the BaP concentration accelerates the degenerate electron exchange (self-exchange) between BaP radical cations and neutral BaP, which becomes faster at higher BaP concentrations and makes the CIDNP cancellation effect more pronounced.^61^ The elementary step can be written as

$${\mathrm{BaP}}^{•+}+\mathrm{BaP}↔\mathrm{BaP}+{\mathrm{BaP}}^{•+}$$

A clear concentration-dependent limitation of the photo-CIDNP effects is observed: at high concentrations of electron donor molecules, hyperpolarization transfer becomes inefficient, leading to a reduction in the photo-CIDNP signal intensity. In biologically relevant systems, however, the analyte concentrations typically fall within the micromolar to nanomolar range, where photo-CIDNP experiments demonstrate higher efficiency.

### Magnetic Field Dependence

^1^H photo-CIDNP NMR spectra of BaP and RF acquired at 400 MHz (9.4 T) and 600 MHz (14.1 T) are shown in Supplementary Figure S8. ^1^H photo-CIDNP spectrum acquired at 400 MHz shows stronger photo-CIDNP BaP signal intensities for protons compared to 600 MHz. In the simplified scenario where all spin–spin interactions are isotropic and exchange is *J*_ex_ = 0, the relevant high-field level-crossing (LC), which turns off coherent evolution of states that only inherit either pure spin-up or spin-down configuration of the ^1^H, occurs when the electron Zeeman frequency difference Δω_e_ = Δ*g*μ_B_ *B* matches half of the effective hyperfine coupling, i.e., at *B*_LC_ ≈ *a*/(2μ_B_|Δ*g*|).^62,63^ This is the classic high-field “matching condition” for Δ*g*-driven S-*T*_0_ mixing in liquids.^64^ Far above this matching field strength (as here, 9.4–14.1 T), Δω_e_ ≫ *a* and coherent S-*T*_0_ mixing becomes less efficient, so the Δ*g* contribution to CIDNP decreases with increasing *B*. Additionally, at higher fields, electron-spin relaxation driven by e.g., the anisotropy of the g-tensor becomes more impactful and dephases the electron-spin coherences,^63^ which may further weaken the spin-sorting efficiency at 14.1 T relative to 9.4 T. These relaxation mechanisms increased population transfer between the eigenstates of the spin system leading to a more uniform overall population and no favoring of specific ^1^H spin states.^63,65^ This mechanism is likely the cause of a decreased photo-CIDNP signal at 14.1 T compared to the 9.4 T case.^63^

### Liquid-State ^1^H Photo-CIDNP NMR of BaP Incorporated DPC Micelles

To investigate whether photo-CIDNP signal enhancement of BaP can be achieved under conditions that more closely resemble physiological environments, the BaP/RF system was incorporated into dodecylphosphocholine (DPC) micelles. DPC micelles are widely used as a well-characterized model for phosphatidylcholine bilayers, the most abundant class of phospholipids in human cell membranes, and can readily incorporate small hydrophilic molecules.^66–68^ For these reasons, DPC was selected as a phosphocholine-like micellar host to solubilize BaP and reproduce a zwitterionic membrane interface.

In the present study, the surfactant concentration was maintained at 15 mM, well above the reported critical micelle concentration (cmc) of ∼1.1 mM for DPC.^69^ Assuming aggregation numbers of 75–80 for DPC micelles,^70^ the BaP concentration of 1 mM corresponds to approximately a 5-fold stoichiometric excess of BaP over micelles. Due to the ring-current effect caused by the delocalized π-electron system of BaP, the NMR signals of DPC protons can be shifted upfield or downfield depending on the orientation and distance of the rings with respect to the protons of the individual DPC segments. The magnitude of the induced chemical shift changes can be used to estimate the average locations of the aromatic cycles in the micelles.

Analysis of the induced chemical shift changes for individual DPC segments (plotted along the long axis of the surfactant molecule; see Figure 4) indicates that BaP is distributed between the hydrophobic core and an interfacial “fence” layer near the F and H segments. The relatively large shifts observed for the F and H groups of DPC lipid protons (Figure 4, Δδ = 0.04 and 0.03 ppm, respectively), compared with those in the hydrophobic core (Δδ ≈ 0.01 ppm), suggest a higher population of BaP in the interfacial region. No chemical shift changes were detected for the headgroup segments E and D of DPC lipid protons, indicating that BaP does not substantially interact with the polar headgroup of DPC. Riboflavin induces much larger and more widespread chemical shift changes for all DPC segments than BaP (Figure 4; Δδ ≈ 0.07–0.11 ppm), indicating a stronger interaction that involves both the hydrophobic chain and the headgroup region. By analogy with other micellar and vesicular systems,^71,72^ this pattern is consistent with a localization of riboflavin in the interfacial region of DPC micelles, between the polar headgroups and the hydrophobic core, although the chemical-shift analysis remains semiquantitative and does not provide a unique structural model.

**Figure 4:**
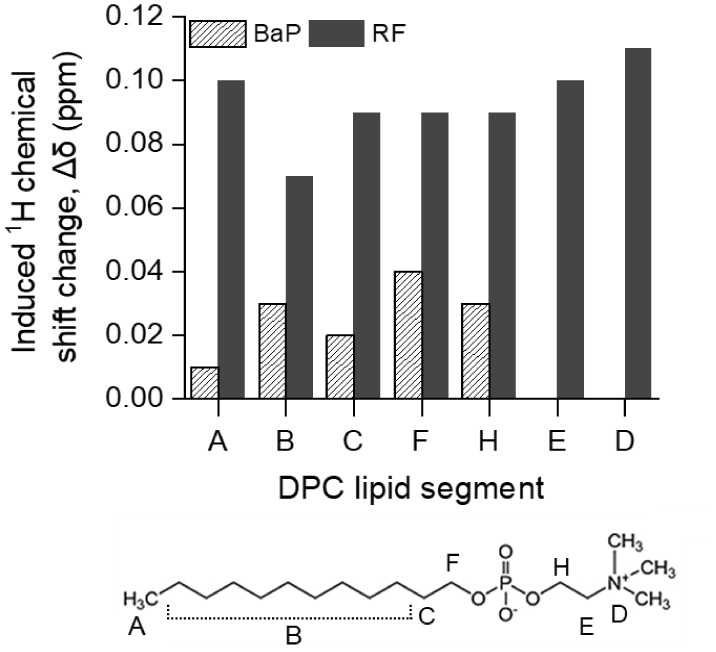
Induced ^1^H NMR chemical shift changes (ppm) of the DPC lipid signals in the presence of BaP and RF, plotted as a function of the lipid segments along the long axis of the DPC molecules in micelles. The structure of the DPC molecule with proton labeling is shown at the bottom.

^1^H photo-CIDNP NMR with BaP and RF system embedded in DPC-*d*_38_ (∼66.66%) micelles was performed at 400 MHz. Figure 5 shows the comparison between photo-CIDNP signal intensities of BaP in the absence (top) and presence (bottom) of DPC micelles. BaP exhibits strong polarization across nearly all proton signals with enhanced absorptive signals in the micellar environment. Embedding BaP and RF into DPC micelles resulted in ∼5-fold enhanced photo-CIDNP signal from BaP as compared to the BaP/RF system in a homogeneous aqueous solution. This enhancement suggests that the lipid phase of DPC micelles facilitates both the formation and stabilization of SCRP, by providing a restricted and partially ordered environment that promotes close proximity and favorable orientation between BaP and RF and limits their relative translational diffusion, thereby prolonging the radical pair lifetime.^73^ The observed upfield shifts of BaP aromatic protons in the presence of DPC micelles indicate an increase in the magnetic shielding. Similar micelle-induced upfield shifts have been reported for other polycyclic aromatic hydrocarbons and aromatic probes in nonionic micelles and are interpreted as signatures of deeper penetration into the surfactant layer or the micellar core.^74–77^

**Figure 5:**
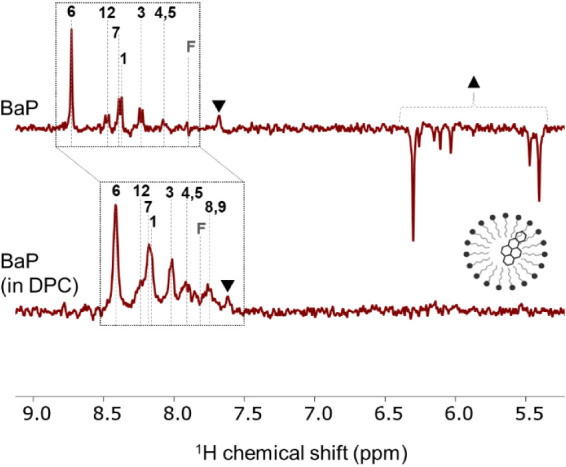
^1^H photo-CIDNP NMR spectra of BaP and RF (top) and BaP and RF incorporated in DPC micelles (bottom), recorded at 400 MHz and 293 K under 445 nm laser illumination (0.9 W, 0.5 s per scan).

The impact of micellar environments on photo-CIDNP enhancement in proteins has been demonstrated in previous studies.^78,79^ Notably, a study on a LytA-derived peptide investigated the influence of increasing DPC micelle concentrations on the photo-CIDNP signal intensity of tyrosine residues, indicating that the micellar microenvironment modulates the local accessibility and dynamic behavior of specific aromatic side chains leading to a micelle induced β-Hairpin-to-α-Helix Transition of a LytA-Derived Peptide.^80^ These findings support the idea that lipid-based assemblies can alter spin dynamics and facilitate radical pair interactions by spatially confining the reacting partners and influencing their orientation and mobility within the micelle. The micellar microenvironment may thus act as a catalytic matrix that promotes more efficient spin polarization transfer, making it a promising model for membrane-assisted hyperpolarization studies in biological systems.

It should be noted that in the photo-CIDNP NMR experiment with surfactant, no formation of oxidized BaP species was detected (Figure 5). It is well established that fatty acids and phospholipids are effective physical quenchers of a singlet oxygen ^1^O_2_,^81,82^ significantly shortening its lifetime in lipid environments compared with bulk D_2_O.^83^ In DPC micelles, both riboflavin and BaP are located in the hydrophobic micellar domain, so singlet oxygen is expected to be generated predominantly within the lipid domain,^84^ where rapid quenching by surrounding acyl chains exceeds diffusion to other targets.^85^

### ^1^H Photo-CIDNP MRI

As a next step, we combined photo-CIDNP-induced signal enhancement with MRI to enable spatially resolved mapping of radical-polarized signals originating from BaP. For this, we applied chemical shift imaging (CSI) in spin echo slab-selective mode to obtain simultaneous spatial and spectral resolution enabling multivoxel spectra over the image. A map of the selected peak provides the spatial distribution of an individual signal over the image. Figure 6 displays images and a matrix array of spectra obtained under illumination of a solution containing BaP (1 mM) and RF (200 μM) in DMSO-*d*_*6*_. Highly resolved spectra were obtained from each voxel, of approximately 18.75 μL. A representative spectrum, taken from the highlighted voxel, where laser light was projecting is shown in Figure 6B. As can be seen from the spectrum, under continuous light irradiation, distinct enhancement of the BaP proton signals (at ^1^H positions 6, 7, 1, and 3) was observed, as compared to the dark condition. The overlaid voxel maps on *T*_2_-weighted RARE image demonstrated a localized increase in signal intensity arising from the proton at position 6 of BaP at the region corresponding to the location of light projection, clearly visible in the CSI color map (Figure 6A center). In contrast, the residual DMSO-*d*_*6*_ signal at 3.36 ppm in the dark condition remained uniform and showed no enhancement, serving as an internal reference for nonphoto-CIDNP effects, leading to the delocalized mapped signal in the whole sample (Figure 6A right). These signals corresponded well with the assigned proton positions of BaP, indicating successful detection and mapping of photoinduced nuclear spin polarization.

**Figure 6:**
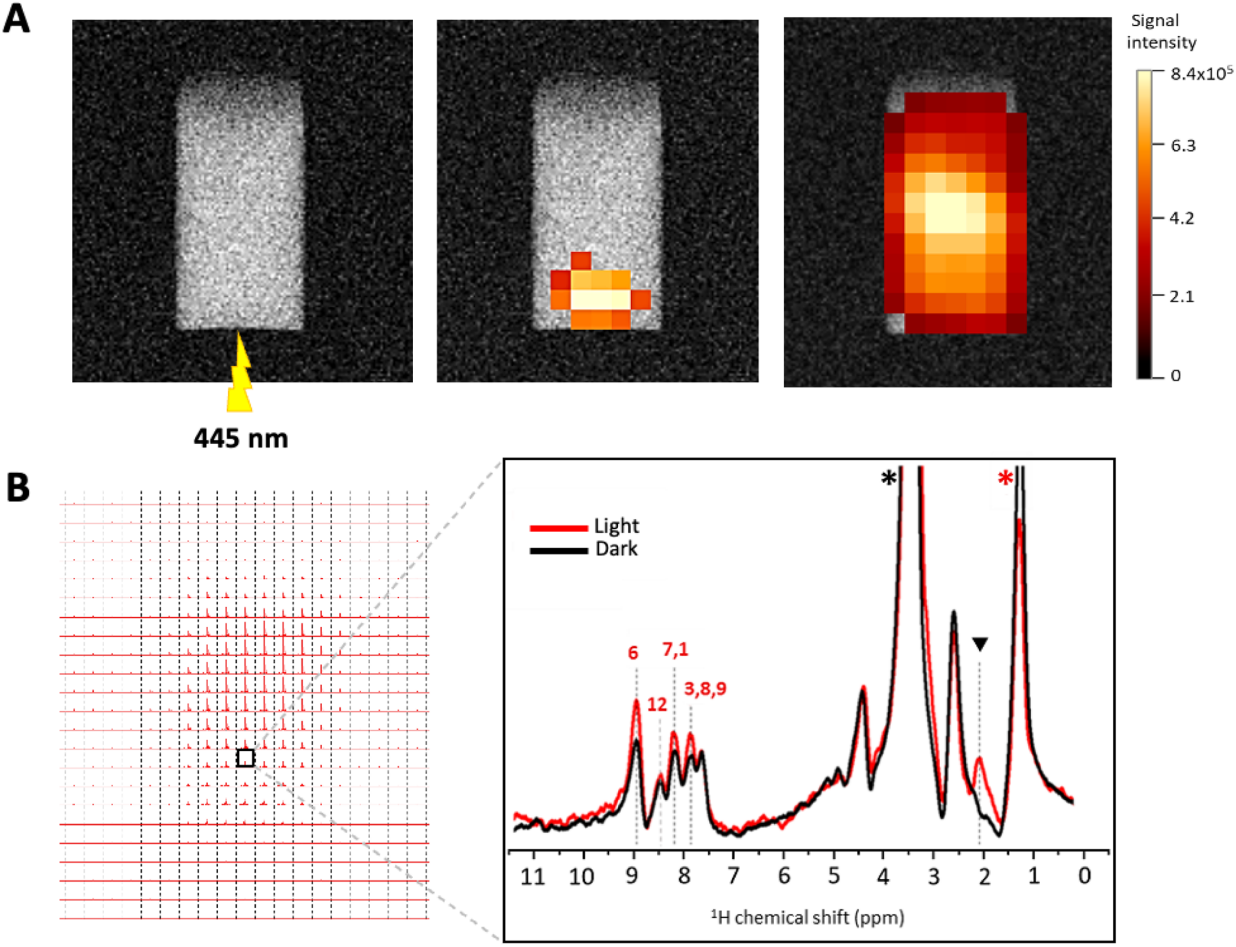
Photo-CIDNP enhanced CSI of a solution containing BaP (1 mM) and RF (200 μM) in DMSO-*d_6_*, recorded at 300 MHz and 293 K. (A) *T*_2_-weighted RARE image and the projection of light (445 nm, 0.6 W, continuous illumination) (left) and corresponding CSI voxel intensity thresholdings of BaP-enhanced signal from position 6 under illumination (center) and a nonphoto-CIDNP signal of residual DMSO-*d*_6_ in dark conditions (right) which were reconstructed as color maps and overlaid on the *T*_2_-weighted RARE image using the Bruker CSI visualization tool. (B) Matrix display of CSI spectra under illumination (left) and spectra from the highlighted voxel (location where light was projected) in magnification in dark and light irradiation are shown in black and red, respectively (right). Signal assignments correspond to the proton positions of BaP and RF. The residual DMSO-*d_6_* signal is marked with a black asterisk. Impurity signal is indicated by a red asterisk at 1.3 ppm. A signal attributed to the photodegradation product appears at 1.9 ppm, marked with a black triangle. CSI data were recorded with a TR = 1000 ms; TE = 15 ms, and a slice thickness was 3 mm. Total averages were 256. Spectral width used was 8 kHz (26.7 ppm), and the 16 × 16 matrix was reconstructed into 32 × 32 voxels.

Our results indicate the potential of CSI combined with photo-CIDNP for spatially resolved mapping of light-activated spin polarization in ^1^H-MRI. The localized signal enhancement observed at highlighted voxels directly correlates with the projected illumination site, indicating that the photo-CIDNP production is spatially confined and highly sensitive to light exposure. The ability to detect photo-CIDNP signal enhancements within such a tiny volume (∼18.75 μL) opens exciting avenues for future applications of photo-CIDNP-enhanced detection of toxins in biological systems by using MRI.

## Conclusions

This study presents the effects of photo-CIDNP in the BaP and RF systems, revealing distinct polarization patterns that were further enhanced in the presence of DPC micelles. The micellar environment facilitated more efficient radical pair interactions, which led to a significant increase in the signal intensities. Hyperfine coupling constants and spin density distributions predicted by DFT calculations identified hydroquinone species as possible oxidized derivatives responsible for the additional emissive peaks observed under illumination. Remarkably, these species were present in extremely low concentrations yet were clearly detected through photo-CIDNP due to its exceptional sensitivity for identifying transient photochemical intermediates. Furthermore, spatial mapping through chemical shift imaging (CSI) provided a unique visualization of the photoinduced nuclear polarization distribution, highlighting the potential of photo-CIDNP MRI as a powerful approach to study light-driven radical mechanisms in biological systems.

## Supplementary Material



## Notes

2D ^1^H–^1^H COSY and 2D ^1^H–^13^C HSQC spectra of BaP, structure and numeration of BaP oxidative products used for DFT calculations, DFT-calculated g-factors and isotropic hyperfine coupling constants *a*_iso_ for BaP and its oxidation products, temporal growth of the signals of oxidative products of BaP over illumination time, comparison of the ^1^H photo-CIDNP NMR spectra of BaP and RF, measured without and with prior N_2_ bubbling, simulated ^1^H NMR spectra of hydroquinone derivatives of BaP, thermally polarized ^1^H NMR spectra of BaP and RF, measured in dark before illumination and in dark after illumination, dependence of ^1^H photo-CIDNP intensities of BaP and BaP_hq_ protons on the BaP:RF molar ratio, and the effect of the magnetic field on ^1^H photo-CIDNP NMR spectra of BaP and RF (PDF)
