# Supplementary material for: Detection of the Carcinogen Benzo[a]pyrene through Photochemically Induced Dynamic Nuclear Polarization: Linking Liquid-State 1H NMR with Spatially Resolved Imaging
Source: Anal Chem. 2026 Apr 29;98(18):13598–608. doi: 10.1021/acs.analchem.6c00224 (PMC13177286; doi:10.1021/acs.analchem.6c00224)
Supplement: Supplementary file 1 [file ac6c00224_si_001.pdf]

Supporting Information

Detection of the Carcinogen Benzo[*a*]pyrene through Photochemically Induced Dynamic Nuclear Polarization: Linking Liquid-State ^{1}H NMR with Spatially Resolved Imaging

Mohd Humair Ali [1,2‡], Guzel Musabirova [1‡], Luca Gerhards [3], Ilia A. Solov'yov [3,4,5], John P. Berry [6], Jörg Matysik[1], A. Alia [2,7*]*

[1]Institut für Analytische Chemie, Universität Leipzig, Linnéstr. 3, D-04103 Leipzig, Germany;
[2]Institut für Medizinische Physik und Biophysik, Universität Leipzig, Härtelstr. 16-18, D-04107 Leipzig, Germany;
[3]Institute of Physics, Carl von Ossietzky Universität, Carl von Ossietzky Str. 9-11, 26129, Oldenburg, Germany;
[4]Center for Nanoscale Dynamics (CENAD), Carl von Ossietzky Universität, Ammerländer Heerstr. 114-118, 26129, Oldenburg, Germany;
[5]Research Centre for Neurosensory Science, Carl von Ossietzky Universität, Carl-von-Ossietzky-Str. 9-11, 26129, Oldenburg, Germany;
[6]Department of Chemistry and Biochemistry, Florida International University, 3000 NE 151st Street, North Miami, FL 33181, USA;
[7]Leiden Institute of Chemistry, Leiden University, Einsteinweg 55, 2301 RA Leiden, The Netherlands
*Corresponding authors. Alia.AliaMatysik@medizin.uni-leipzig.de (A.A); Joerg.Matysik@uni-leipzig.de (J.M.)

Table of Contents for Supporting Information

Standard NMR measurements ……………………………………………………………S2

2D ^{1}H-^{1}H COSY and 2D ^{1}H-^{13}C HSQC spectra of BaP ...……………………………….…S3

Structure and numeration of BaP oxidative products used for DFT calculations …………...S4

DFT-calculated g-factors and isotropic hyperfine coupling constants a_{iso} for BaP and its oxidation products...S5

Temporal growth of the signals of oxidative products of BaP over illumination time ……...S6

Comparison of the ^{1}H photo-CIDNP NMR spectra of BaP and RF, measured without and with prior N_2 bubbling...S7

Simulated ^{1}H-NMR spectra of hydroquinone derivatives of BaP.......................................S8

Thermally polarized ^{1}H NMR spectra of BaP and RF, measured in dark before illumination and in dark after illumination...S9

Dependence of ^{1}H photo-CIDNP intensities of BaP and BaP_{hq} protons on the BaP: RF molar ratio...S10

Effect of the magnetic field on ^{1}H photo-CIDNP NMR spectra of BaP and RF..................S11

Standard NMR measurements

Standard NMR experiments were performed on an AvanceNeo 400 NB spectrometer (Bruker BioSpin GmbH) equipped with a 5 mm BBO probe. All measurements were conducted at 293 K. Thermally polarized ^{1}H spectra were acquired using the standard zg30 pulse sequence. Two-dimensional ^{1}H–^{13}C HSQC spectra were recorded with the gradient-selected hsqcetgp pulse program. Typical acquisition parameters were: 512 complex points (^{1}H) × 256 complex points (^{13}C), relaxation delay of 3 s, and 128 scans per increment. Two-dimensional ^{1}H–^{1}H COSY spectra were obtained using the cosygpppqf pulse sequence with 1024 × 128 complex points, 152 scans per increment, and a relaxation delay of 2 s. For all NMR measurements, chemical shifts were referenced to the residual solvent signals (δ_H = 2.50 ppm, δ_C = 39.5 ppm for DMSO-d_6).

For data processing (phase correction, baseline correction), as well as data evaluation, TOPSPIN 4.0.6 (Bruker Biospin GmbH, Germany) and MestreNova version 14.1.0-24037 (Mestre lab Research S.L.) were used.

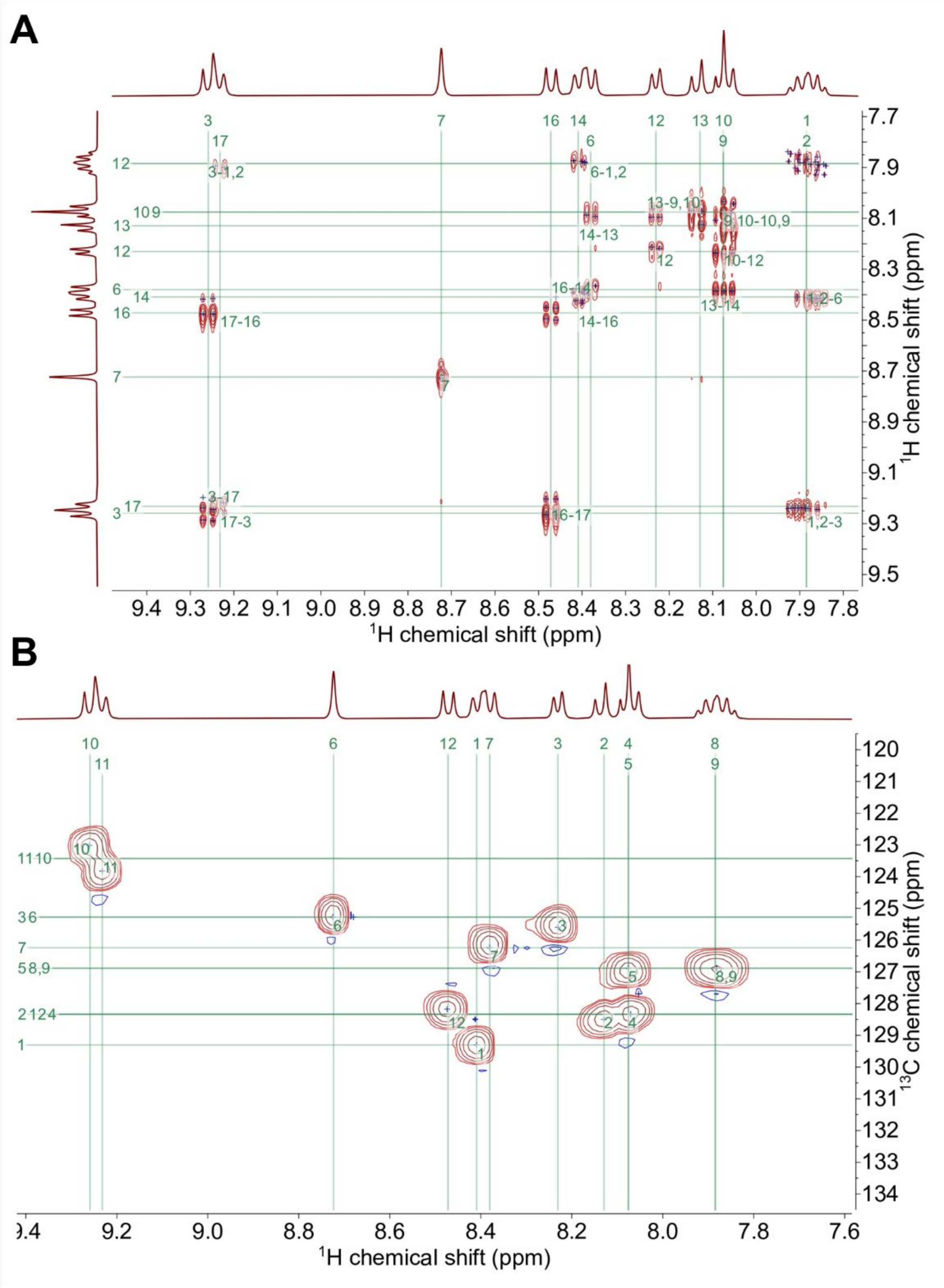

Figure S1. 2D ^{1}H-^{1}H COSY (A) and 2D ^{1}H-^{13}C HSQC (B) spectra of BaP (10 mM) in DMSO-d_6, recorded at 400 MHz and 293 K.

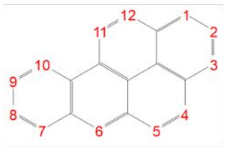

Benzo[*a*]pyrene

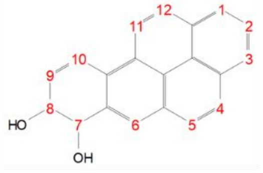

Benzo[*a*]pyrene-7,8-diol

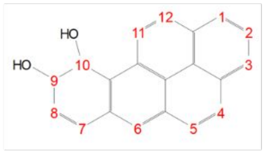

Benzo[*a*]pyrene-9,10-diol

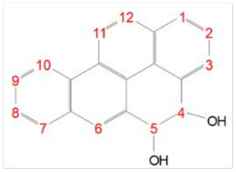

Benzo[*a*]pyrene-4,5-diol

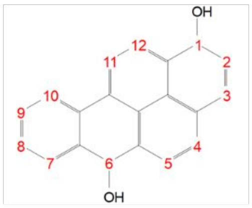

Benzo[*a*]pyrene-1,6-hydroquinone

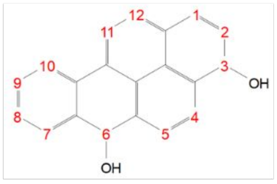

Benzo[*a*]pyrene-3,6-hydroquinone

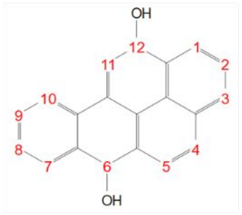

Benzo[*a*]pyrene-6,12-hydroquinone

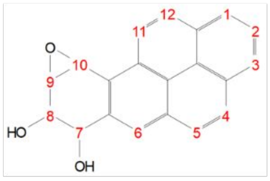

Benzo[*a*]pyrene-7,8-diol-9,10-oxide

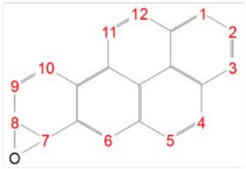

Benzo[*a*]pyrene-7,8-oxide

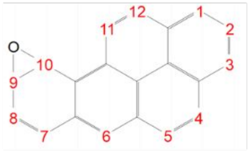

Benzo[*a*]pyrene-9,10-oxide

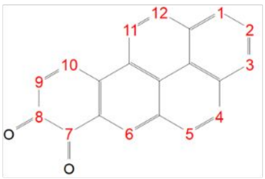

Benzo[*a*]pyrene-7,8-dione

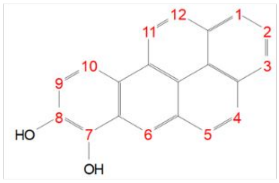

Benzo[*a*]pyrene catechol

Figure S2. Structure and numeration of BaP oxidative products used for DFT calculations.

Table S1. DFT-calculated g-factors and isotropic hyperfine coupling constants a(iso) for BaP and its oxidation products.

Compounds	BaP	BaP-7,8-diol	BaP-9,10-diol	BaP-4,5-diol	**BaP-1,6-hydroquinone**	**BaP-3,6-hydroquinone**	**BaP-6,12-hydroquinone**	BaP-7,8-diol-9,10-oxide	BaP-7,8-oxide	BaP-9,10-oxide	BaP-7,8-dione	BaP catechol
g-factor	2.0026	2.0026	2.0026	2.0026	2.0024	2.0022	2.0023	2.0026	2.0029	2.0029	2.0029	2.0028
Proton	*a*(iso), MHz											
1	-13.92	-13.15	-15.73	-8.60	**104.08**	3.31	-5.35	-15.43	-13.67	-15.92	-13.77	-9.30
2	3.34	4.84	4.45	0.87	-12.13	-15.91	0.93	4.52	4.15	4.48	3.98	1.72
3	-11.40	-15.45	-15.99	-5.66	2.29	**76.72**	-8.41	-15.90	-14.02	-15.65	-14.62	-6.93
4	-5.16	-8.56	-5.91	5.97	-3.01	-2.08	-13.37	-6.03	-7.25	-5.77	-5.35	-3.98
5	-5.45	-0.71	-6.13	2.09	-4.46	-1.93	-0.80	-5.87	-3.10	-6.29	-5.41	-3.64
6	-19.49	-8.00	-15.53	-16.20	**63.67**	**48.18**	19.86	-14.82	-11.12	-16.13	-12.73	-17.33
7	-8.72	-3.66	-1.97	-8.60	1.58	2.04	-0.34	-1.41	2.99	-1.87		
8	2.28	28.29	2.47	0.87	-5.18	-7.14	-1.42	1.99	0.79	3.77		
9	-7.74	-17.72	-0.16	-5.71	1.28	1.82	0.38	4.36	-15.48	3.35	-13.47	1.80
10	-0.68	5.95	7.34	-4.01	-3.08	-4.44	-0.92	0.28	4.39	0.04	6.42	-7.85
11	0.12	-3.82	-6.60	-0.83	-2.87	-5.69	-24.65	-6.24	-3.87	-5.64	-6.86	1.99
12	-8.39	-5.35	-5.23	-15.35	-4.06	-3.39	**128.25**	-5.39	-5.94	-5.89	-3.95	-6.88
7-OH		0.23						-1.41				-4.68
8-OH		0.01						1.99				-2.31
9-OH			0.17									
10-OH			5.36									
4-OH				-0.02								
5-OH				0.18								
1-OH					-1.14							
3-OH						0.04						
6-OH					0.44	0.42	-0.09					
12-OH							1.71					

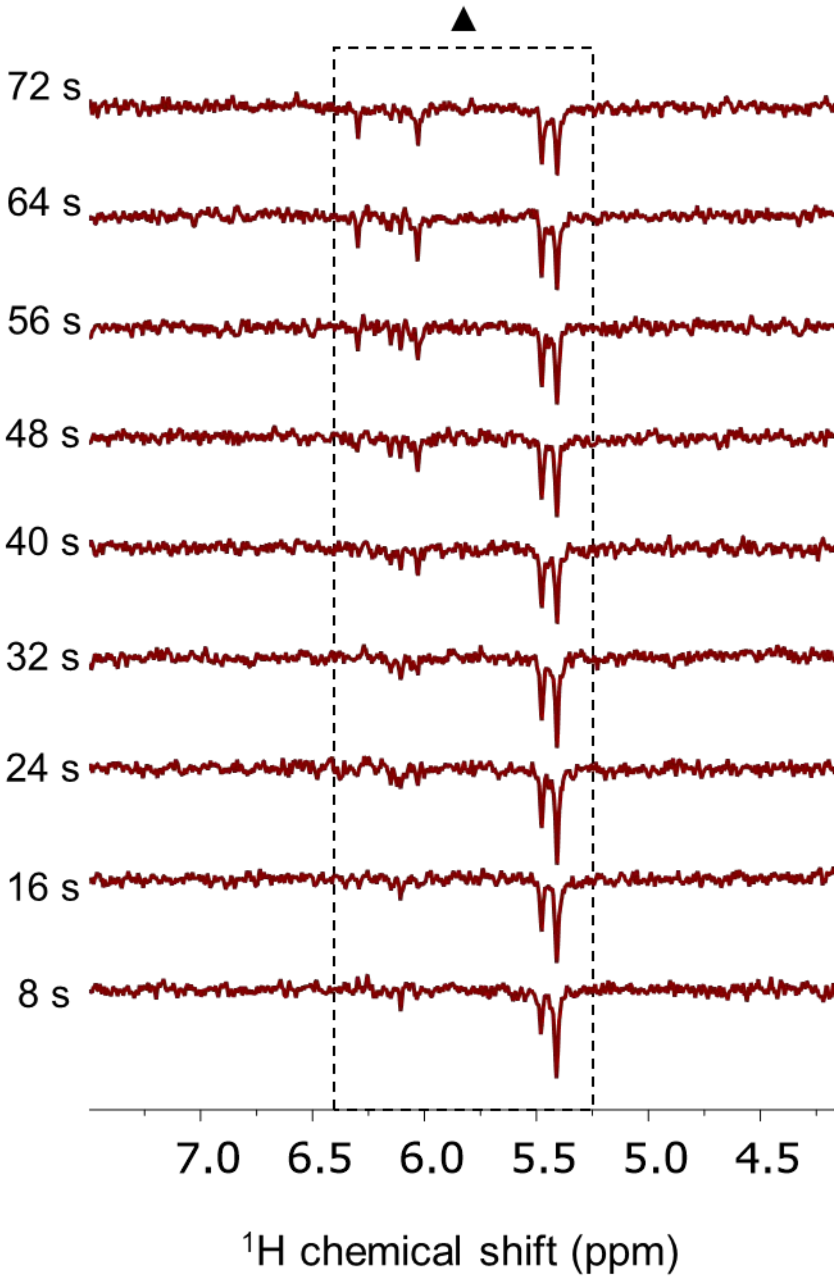

Figure S3. ^{1}H Photo-CIDNP NMR spectra showing temporal growth of the signals of oxidative products of BaP (triangle) over illumination time @9.4T, 293 K, 445 nm at 0.9W for 0.5 s.

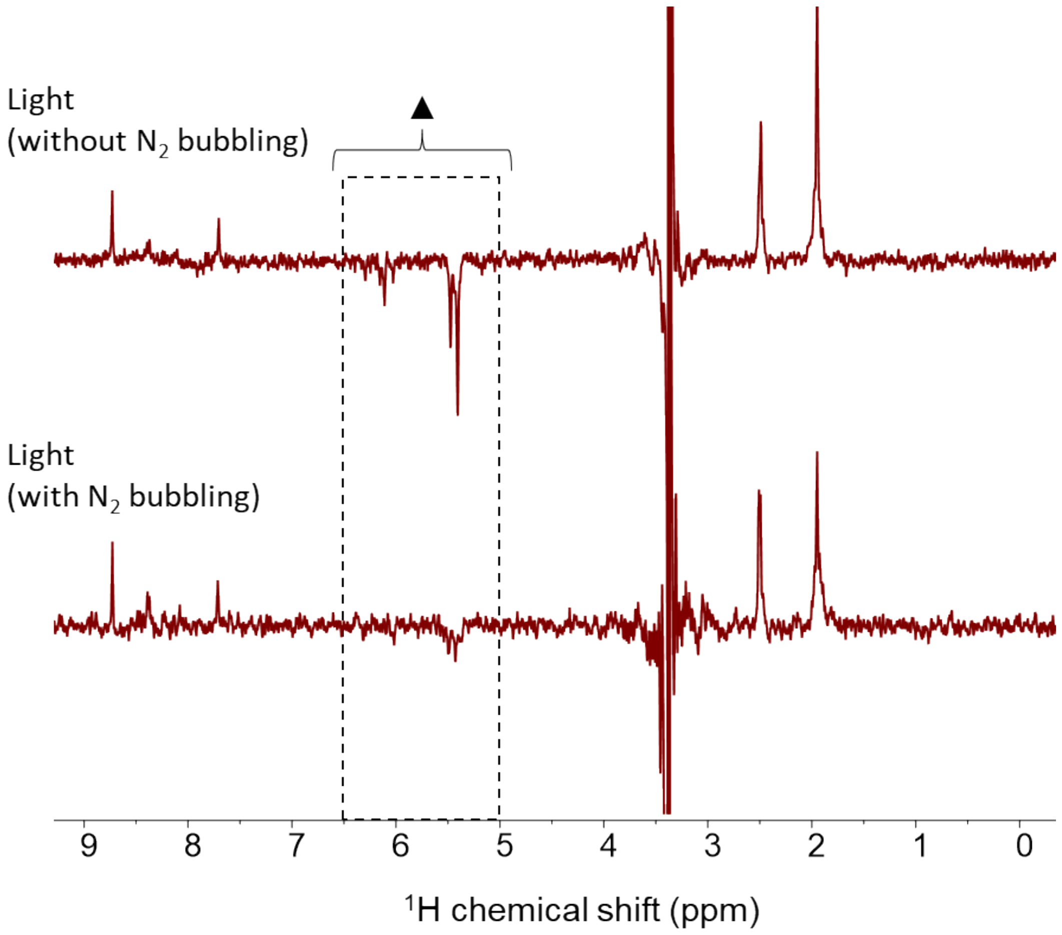

Figure S4. Comparison of the ^1H photo-CIDNP NMR spectra of solutions of BaP (1 mM) and riboflavin (200 μM), measured without (upper) and with (lower) prior N_2 bubbling to remove oxygen**.** As shown, the signals (triangle) corresponding to the oxidative products of BaP (triangle) were present in the upper spectrum but almost disappeared in the samples bubbled with N_2 to remove oxygen.

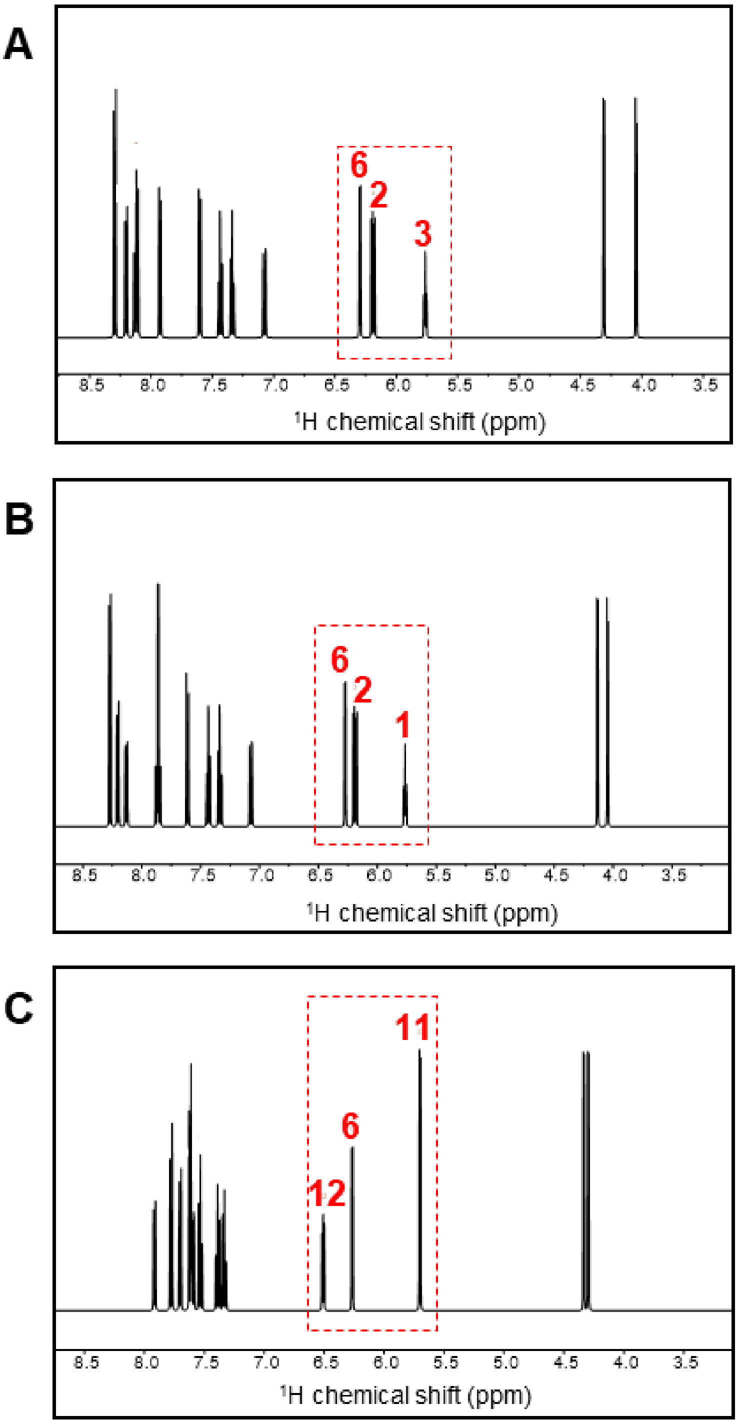

Figure S5 Simulated ^{1}H-NMR spectra of Benzo[a]pyrene-3,6-hydroquinone (A), Benzo[a]pyrene-1,6-hydroquinone (B) and Benzo[a]pyrene-6,12-hydroquinone (C) in DMSO obtained from MestreNova (version 14.2.1). Protons at positions 6, 3 and 1 of BaP hydroquinones are predicted to exhibit negative (emissive) photo-CIDNP signals and they also match with the chemical shifts of photo-CIDNP region ($\delta \approx 6.3$ ppm and 5.4 ppm), which is in agreement with the experimentally observed polarization pattern.

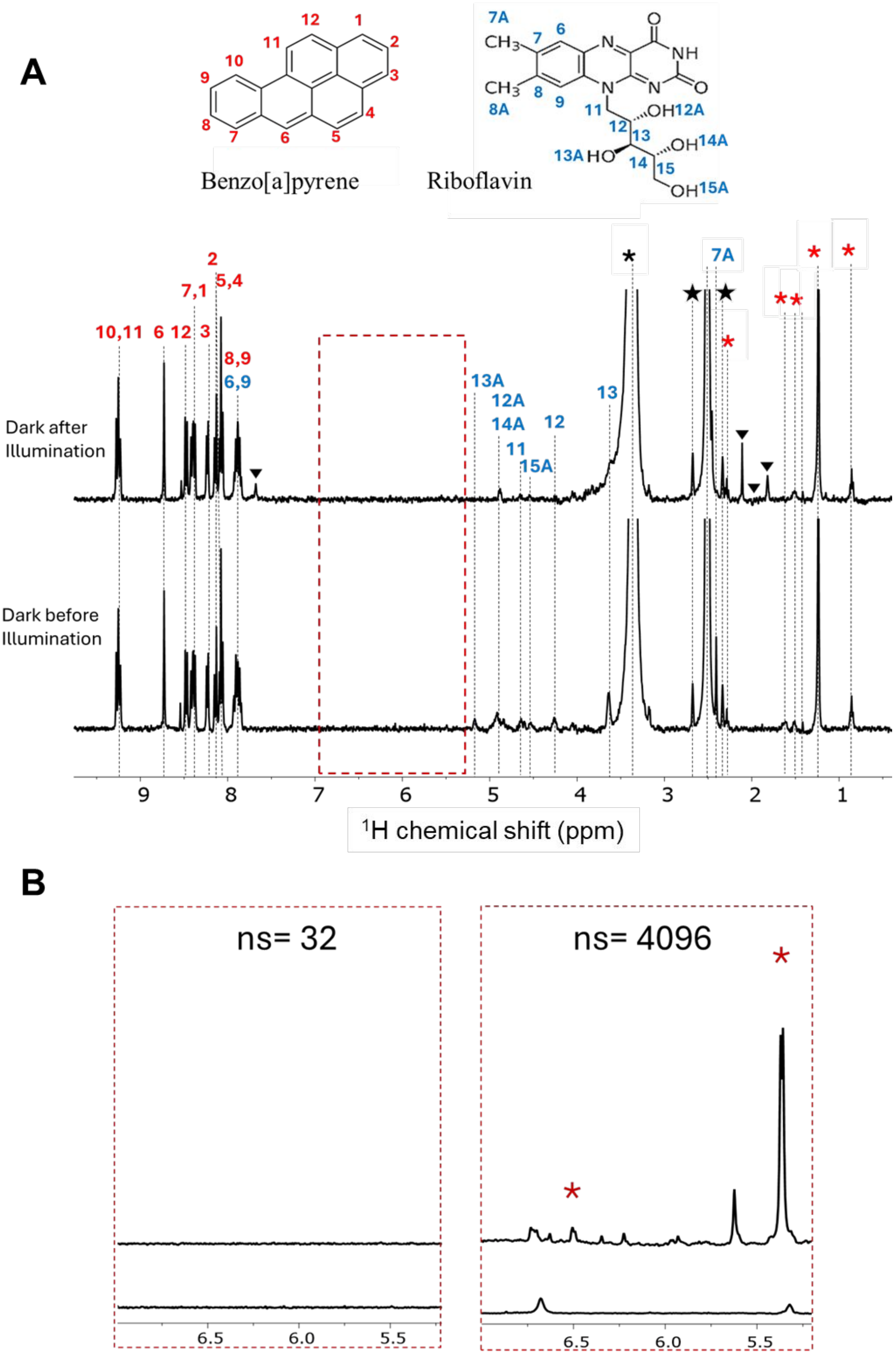

Figure S6 Thermally polarized ^{1}H NMR spectra of solutions of BaP (1 mM) and riboflavin (200 µM), measured in dark before illumination (bottom) and in dark after illumination (top) using conventional single pulse (zg) sequence. (A) Measured with number of scans (ns)=32; (B) Comparison between ns=32 and ns=4096.

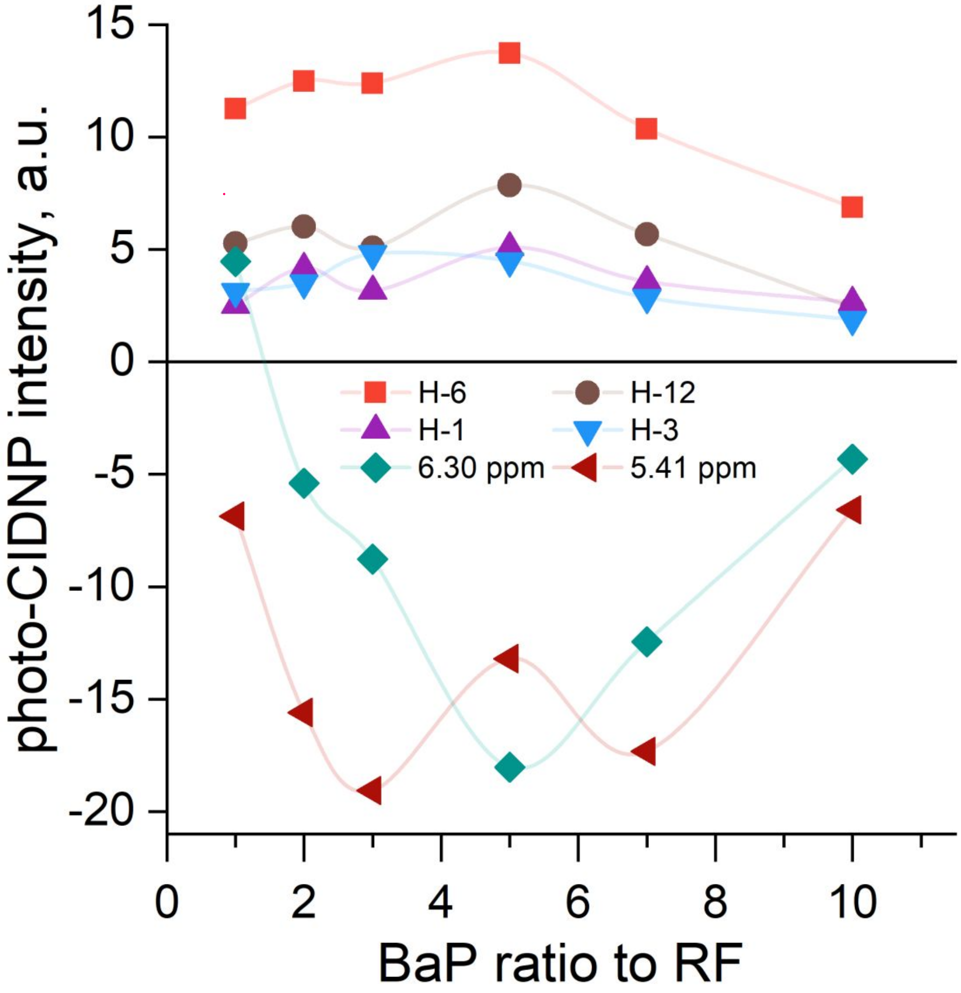

Figure S7. Dependence of ^{1}H photo-CIDNP intensities of BaP and BaP_{hq} protons on the BaP:RF molar ratio. Positive and negative signals correspond to emissive and absorptive proton responses, respectively.

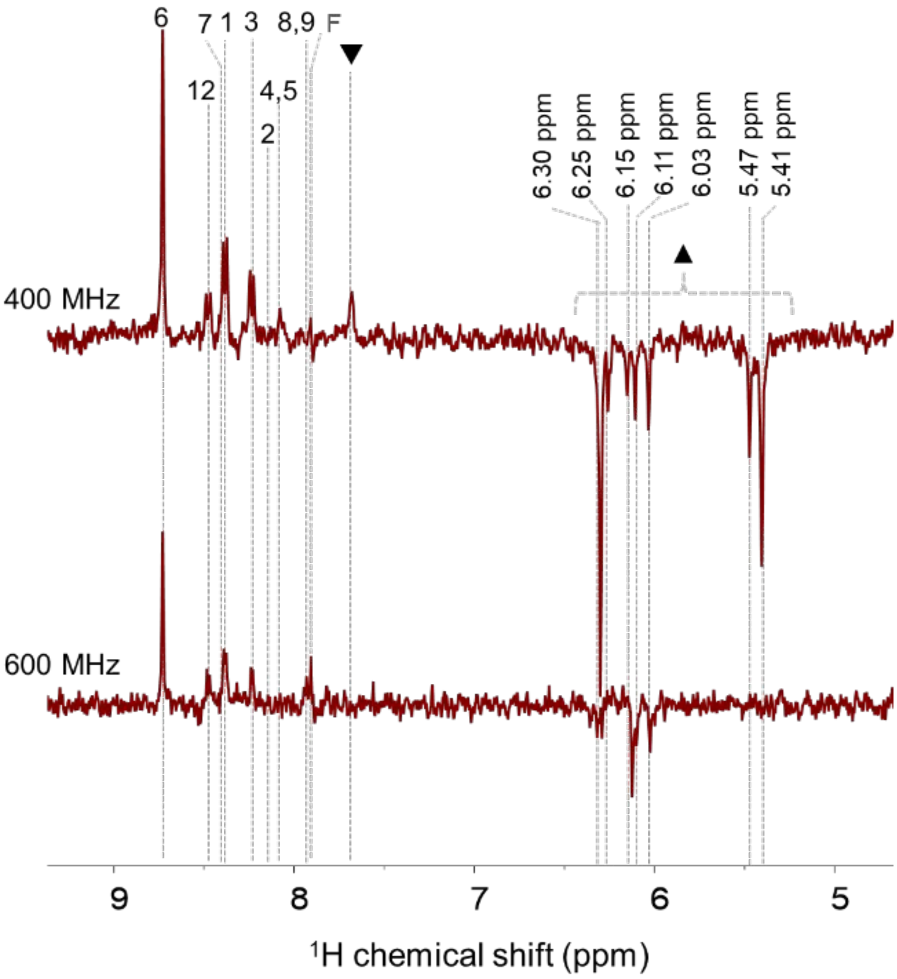

Figure S8. Effect of the magnetic field on ^{1}H photo-CIDNP NMR spectra of BaP and RF (molar ratio 5:1) in DMSO-d_6, recorded at 600 MHz (bottom) and 400 MHz (top) and 293 K under 445 nm laser illumination (0.9 W, 0.5 s per scan). Signal assignments correspond to the proton numbering in the molecular structure of BaP (see Fig. 1). Signal attributed to photodegradation product of RF appears at 7.7 ppm and is marked with down-triangles (▼). Signals at 5.4 - 6.3 ppm, observed exclusively under illumination from oxidative product of BaP, are marked with up-triangle (▲).
